# Can a Patient’s In-Hospital Length of Stay and Mortality Be Explained by Early-Risk Assessments?

**DOI:** 10.1371/journal.pone.0162976

**Published:** 2016-09-15

**Authors:** Nasibeh Azadeh-Fard, Navid Ghaffarzadegan, Jaime A. Camelio

**Affiliations:** 1 Industrial and Systems Engineering Department, Rochester Institute of Technology, Rochester, New York, United States of America; 2 Grado Department of Industrial and Systems Engineering, Virginia Tech, Blacksburg, Virginia, United States of America; University College London, UNITED KINGDOM

## Abstract

**Objective:**

To assess whether a patient’s in-hospital length of stay (LOS) and mortality can be explained by early objective and/or physicians’ subjective-risk assessments.

**Data Sources/Study Setting:**

Analysis of a detailed dataset of 1,021 patients admitted to a large U.S. hospital between January and September 2014.

**Study Design:**

We empirically test the explanatory power of objective and subjective early-risk assessments using various linear and logistic regression models.

**Principal Findings:**

The objective measures of early warning can only weakly explain LOS and mortality. When controlled for various vital signs and demographics, objective signs lose their explanatory power. LOS and death are more associated with physicians’ early subjective risk assessments than the objective measures.

**Conclusions:**

Explaining LOS and mortality require variables beyond patients’ initial medical risk measures. LOS and in-hospital mortality are more associated with the way in which the human element of healthcare service (e.g., physicians) perceives and reacts to the risks.

## Introduction

Risk evaluation and control are important components of healthcare operations. Ideally, providers would like to be able to predict health risks early in hospital admissions to take subsequent controlling actions. Different methods and techniques have been developed for this purpose, one of which is the early warning system. An early warning system is a measurement tool to assess patient health risks objectively and to quickly determine the degree of illness [1].

Several major early warning systems exist and each type offers a slight variation in risk- assessment parameters. The Modified Early Warning Score (MEWS) [2], the focus in our study, is a commonly used triage tool to quickly determine the severity of a hospitalized patient’s illness [2–5]. The MEWS system utilizes five major vital signs—systolic blood pressure, pulse rate, respiratory rate, temperature, and the AVPU score (A: alert, V: responding to voice, P: responding to painful stimuli, U: unresponsive)—to assign an aggregate number to each patient, which comprises the patient’s health risk. Obtaining a MEWS involves assigning a number between 0 and 3 to each of the 5 vital signs. The sum of the 5 numbers yields the patient’s total MEWS score—between 0 and 15. A total score of four or higher prompts a nurse to call the patient’s physician or the hospital’s rapid response team (for more information, see reference [4]). Other early warning systems follow a similar logic. The Standardized Early Warning System (SEWS) [6] adds oxygen saturation level (SpO2%) to the five MEWS vital signs to detect a patient’s deterioration. The Decision-Tree Early Warning Score (DTEWS) [7] is a decision-tree analysis based on a database of vital signs. The National Early Warning Score (NEWS) [8–11] utilizes seven variables to identify deteriorating patients: respiratory rate, oxygen saturation, any supplemental oxygen, temperature, systolic blood pressure, heart rate, and level of consciousness.

The main use of early warning scores is to provide early warnings to health providers to spur quick, preventive reactions. Some experts have argued that these measures in fact have much more to offer, such as helping to predict patients’ length of stay (LOS) in hospitals or health outcomes—e.g., the chance of in-hospital mortality [4, 6, 12, 13]. Given the importance of LOS and mortality in assessing healthcare quality, resource allocations, and costs [14], the hope is that early warning scores can make it possible to explain and improve hospital utilization and outcomes.

While a warning system can be an efficient and rapid way of reducing or preventing life-threatening events, there is mixed evidence regarding the effectiveness of early warning scores. On the one hand, MEWS can predict an increased risk of death or admission to an intensive care unit (ICU) or high dependency unit (HDU) [2]. MEWS can also be used to identify patients who need hospital admission as well as those at higher risk of in-hospital death [12]. The proportion of patients admitted and who subsequently died in the hospital was significantly higher for higher MEWS values [12]. SEWS have also been correlated with a patient’s LOS [6]. On the other hand, some studies have suggested that further work is needed to derive and validate early warning scores, and that scores that utilize inappropriate parameters and cut-off points should be replaced with those with higher diagnostic accuracy [15–18].

A potential limitation to these and many other similar studies is a lack of detailed administrative-level data on patient and physician characteristics. Some of these studies focus on assessing simple correlations between two variables without controlling for variation across patients or across physicians. Thus, whether early warning scores influence physicians’ diagnoses and risk assessments remains an open question. In other words, we do not know if physicians are actually using these measures and if such measures are preferred over early subjective assessments.

In this study, we assess whether a patient’s admission MEWS and vital signs are associated with LOS and in-hospital mortality. We also compare and contrast MEWS as an objective measure with physicians’ subjective-risk assessments early after admission.

## Methodology

We collected detailed data for 1,021 randomly selected patients admitted between January and September 2014 to an over-500-bed medical center (Lewis Gale Medical Center) in the state of Virginia. Our study was approved by the Lewis Gale Medical Center Education Department as patient records were anonymized and de-identified prior to analysis and the dataset is considered exempt by Virginia Tech’s Institutional Review Board. Patient records were accessed through the electronic patient management systems (MEDITECH and Crimson), which contain full patient demographic data along with individual records and medical documents including various vital signs, MEWS, clinicians’ early assessments of severity level and mortality risk, and patient outcomes such as hospital LOS and disposition conditions. Physicians’ identifiers were included in the data. Newborns and patients admitted to the ICU were excluded from our study. The data is available in S1 Data file.

Table 1 shows the descriptive statistics for some of the main variables in our analysis that are stratified by dead versus survivors. Severity level and mortality risk are categorical variables between 1 and 4, where 1 represents the lowest severity level or mortality risk. In addition, we controlled for gender, weight, height, BMI, physician, and additional physiological measurements including oxygen saturation level and diastolic blood pressure (DBP). The variable physician is a nominal variable assigned to each attending physician who makes the subjective measures. AVPU is a categorical and nominal variable with four values (A, P, V, and U).

**Table 1:** Descriptive Statistics of Variables.

	Survivors (N = 985)				Dead (N = 36)			
Variable	Mean	Std Dev	Min	Max	Mean	Std Dev	Min	Max
**Severity Level (1 to 4)**	2.17	0.84	1	4	3.39	0.77	1	4
**Mortality Risk (1 to 4)**	1.85	0.90	1	4	3.39	0.87	1	4
**Length of Stays (Days)**	4.70	4.20	1	31	4.94	6.17	1	30
**MEWS**	1.55	1.09	0	8	2.89	2.19	0	9
**Temperature**	97.81	3.57	33.70	103.20	94.10	14.53	34	100.10
**Pulse Rate**	82.41	19.09	20	160	88.72	18.17	48	128
**Respiratory Rate**	18.36	2.88	9	44	20.39	5.98	8	42
**SBP**	136.23	24.51	79	243	128.75	29.33	80	213
**Age**	66.22	16.92	18	101	75.25	15.01	30	94

Table A1 in S1 Appendix reports the variables and correlations between any pairs of the variables. We found no serious correlation between the variables other than between two subjective assessments of physicians—that is, physicians’ assessment of mortality risk and severity level, which was expected.

We investigated associations between different subjective and objective measures and our outcome variables. Our analysis included a range of stepwise regressions. In this study, and in the interest of parsimony, we focused on two main models: an ordinary least square, fixed-effect model to explain LOS, and a logistic regression model to explain mortality; both models were based on a wide range of independent and control variables. In addition, we analyzed effects of vital signs and early-risk indicators on physicians’ early subjective-risk assessments.

## Results

Table 2 summarizes the results of our stepwise regression analysis for LOS in which different independent variables were controlled in each step. In addition to the presented variables, patient demographics were controlled. Although the first column in Table 2 shows that the MEWS’s coefficient is significant, the low value of R^2^ (0.02) depicts that MEWS is only slightly better than a random guess for explaining LOS, This means that the whole model only predicted 2% of LOS variation.

**Table 2:** Regression Analysis Results for LOS.

Source (*x*_*i*_)	M1	M2	M3	M4	M5	M6	M7
*Patient Demographics*				controlled	controlled	controlled	
MEWS	0.57** (0.11)	0.27 (0.19)	0.28 (0.19)	0.30 (0.19)	0.16 (0.18)	0.26 (0.18)	0.07 (0.11)
*Vital Signs*							
Temperature		0.1*** (0.03)	0.09*** (0.03)	0.09*** (0.03)	0.07** (0.03)	0.06 (0.03)	
Pulse Rate		0.02** (0.01)	0.03*** (0.01)	0.03*** (0.01)	0.01 (0.01)	0.01 (0.01)	
Respiratory		0.01 (0.05)	0.01 (0.05)	-0.001 (0.05)	-0.04 (0.05)	-0.05 (0.05)	
SBP		-0.01* (0.01)	0.00 (0.01)	-0.004 (0.01)	0.002 (0.01)	0.01 (0.01)	
AVPU		not significant	not significant	not significant	not significant	not significant	
*Additional Physiological Measures*							
DBP			-0.03*** (0.11)	-0.02 (0.01)	-0.01 (0.01)	-0.01 (0.10)	
SpO2%			0.03 (0.05)	0.05 (0.05)	0.05 (0.04)	0.05 (0.04)	
*Subjective Assessments*							
Severity Level					1.76*** (0.22)	1.77*** (0.23)	1.81** (0.21)
Mortality Risk					0.47** (0.21)	0.53** (0.22)	0.37** (0.19)
Physician						significant	
Intercept	3.80*** (0.22)	-4.58 (3.25)	-6.55 (0.25)	-25.21** (10.34)	-27.02*** (9.52)	-26.22*** (9.92)	-0.10 (0.34)
*R*^2^	0.02	0.05	0.05	0.06	0.21	0.33	0.19
*R*^2^ adjusted	0.02	0.04	0.04	0.05	0.20	0.24	0.19
Observations	1010	1010	1010	1010	1010	1010	1010

***p < 0.01,

**p < 0.05,

*p < 0.1

Note: Standard errors are in parentheses.

In models M2–M6, however, MEWS is no longer significant. This is not unexpected, and one may argue that it does not speak against the usefulness of MEWS. We point out two major observations: (1) the model with only MEWS as a control variable has a very low predictive power (*R*^2^) even though MEWS is significant. This resonates with the common statistical argument that “significance” is different than “meaningfulness”: MEWS is significant in M1, but model M1 is only slightly better than a random guess; (2) as shown in the results of M2–M6, not all variables within MEWS (i.e., vital signs) are significant. In other words, better warning scores can be constructed based on weighted scores of some but not all of the vital signs. In models M5 and M6, vital signs lose their significance. In this step-wise analysis, R^2^ increases from 0.02 in M1 to 0.33 in model 6.

Finally, M7 shows the results of a model with the subjective measures and MEWS (but not the components of MEWS) in which MEWS is not a significant variable.

In sum, as shown in the table, in more complete models, none of the MEWS or vital signs at the time of admission is associated with LOS. However, physicians’ subjective assessments represented by their assessment of mortality risk and severity level are significantly associated with LOS. We later analyze how physicians make subjective assessments.

We now look at the mortality outcome variable. Table 3 shows the results of our mortality analysis with logistic regressions and reports the odds ratios. In model M1, MEWS is positively associated with mortality. In models M2–M5, we find that in controlling for more variables, MEWS loses its statistical significance and that the direction of association is negative in M3–M5. Physicians’ subjective assessments seem to have a higher explanatory power of mortality. Specifically, physicians’ subjective assessment of mortality risk is significantly and positively associated with the actual mortality. This finding leads to the conclusion that patients with higher mortality risk values at the time of admission are more likely to die. In another model, we added “physician” as a control variable in our analysis, but it was not significantly associated with mortality.

**Table 3:** Logistic Regression Analysis Results for Mortality.

Source (*x*_*i*_)	M1	M2	M3	M4	M5	M6
*Patient Demographics*				controlled	controlled	
MEWS	1.68***	1.13	0.96	0.87	0.80	1.21*
	(1.41–2.02)	(0.72–1.73)	(0.60–1.50)	(0.53–1.37)	(0.46–1.33)	(0.98–1.48)
*Vital Signs*						
Temperature		0.98	0.98	0.96*	0.94*	
		(0.95–1.02)	(0.94–1.01)	(0.92–1.00)	(0.89–1.00)	
Pulse Rate		1.00	1.01	1.01	1.01	
		(0.99–1.03)	(0.99–1.03)	(0.99–1.04)	(0.98–1.03)	
Respiratory		1.09* (0.99–1.21)	1.11* (1.00–1.24)	1.15** (1.03–1.28)	1.12* (0.99–1.26)	
SBP		0.99	1.00	0.99	1.00	
		(0.98–1.01)	(0.98–1.01)	(0.97–1.01)	(0.98–1.02)	
AVPU		significant	significant	not significant	not significant	
*Additional Physiological Measures*						
DBP			0.99	1.00	1.01	
			(0.96–1.02)	(0.97–1.04)	(0.98–1.05)	
SpO2%			0.88*** (0.80–0.96)	0.88** (0.80–0.97)	0.90* (0.81–1.00)	
*Subjective Assessment*						
Severity Level					2.21*	1.49
					(0.96–5.28)	0.71–3.22)
Mortality Risk					3.89*** (1.87–8.55)	3.93** (2.00–8.19)
LOS					0.88** (0.79–0.96)	
C-Statistic	0.68	0.75	0.75	0.94	0.97	0.89
Log Likelihood	-141.46	-136.80	-132.60	-85.91	-62.24	-109.80
Observations	1010	1010	1010	1010	1010	1010

***p < 0.01,

**p < 0.05,

*p < 0.1;

Note: The numbers out of parenthesis show the odds ratios and 95% confidence intervals are inside parenthesis; an odds ratio less than 1 represents a smaller likelihood of mortality.

The results show that LOS is significantly and negatively associated with mortality; that is, the probability of mortality decreases the longer a patient stays in the hospital. It should be noted that, this can also be due to a reverse causality effect meaning that people who survive tend to stay longer in the hospital. As previously stated, no serious correlation exists between any pairs of variables in our analysis other than the correlation between the two subjective measures (Table A1).

In short, although both analyses show that using MEWS as the only independent variable can somewhat explain LOS and death, these models are weak and only slightly better than random guesses. If more variables are added, it better explains LOS and death. Both of the models point to the importance of physicians’ subjective assessments. It is important to look at the effects of different variables, including vital signs, demographic characteristics, and MEWS on physicians’ subjective assessments of severity level and mortality risks. The results are shown in Tables 4 and 5, which demonstrate that adding vital signs make a stronger model. In other words, it seems that physicians look at some of the vital signs (especially pulse rate, SBP, and AVPU) rather than the aggregate measure of MEWS in assigning risk measures.

**Table 4:** Regression Analysis Results for Physician’s Subjective Assessments (severity level).

Source (*x*_*i*_)	M1	M2	M3	M4	M5
*Patient Demographics*				controlled	controlled
MEWS	0.22*** (0.02)	0.08** (0.04)	0.07** (0.04)	0.06 (0.04)	0.03 (0.03)
*Vital Signs*					
Temperature		0.1** (0.01)	0.01** (0.01)	0.01* (0.01)	0.01 (0.01)
Pulse Rate		0.01*** (0.002)	0.01*** (0.002)	0.01*** (0.001)	0.01*** (0.002)
Respiratory		0.02** (0.01)	0.02* (0.01)	0.02* (0.01)	0.02* (0.01)
SBP		-0.004*** (0.001)	-0.001 (0.001)	-0.002** (0.001)	-0.004*** (0.001)
AVPU		significant	significant	significant	significant
*Additional Physiological Measures*					
DBP			-0.01*** (0.002)	-0.01*** (0.002)	-0.01** (0.002)
SpO2%			-0.01 (0.01)	-0.002 (0.01)	-0.003 (0.01)
Physician					significant
Intercept	1.86*** (0.04)	0.84 (0.62)	1.95 (1.07)	1.48 (1.92)	1.74 (1.92)
*R*^2^	0.09	0.15	0.16	0.20	0.38
*R*^2^ adjusted	0.09	0.14	0.16	0.19	0.30
Observations	1010	1010	1010	1010	1010

***p < 0.01,

**p < 0.05,

*p < 0.1

Note: Standard errors are in parentheses.

**Table 5:** Regression Analysis Results for Physicians’ Subjective Assessment (mortality risk).

Source (*x*_*i*_)	M1	M2	M3	M4	M5
*Patient Demographics*				controlled	controlled
MEWS	0.24*** (0.02)	0.11** (0.04)	0.11*** (0.04)	0.08** (0.04)	0.06 (0.04)
*Vital Signs*					
Temperature		0.02*** (0.01)	0.01** (0.01)	0.01** (0.01)	0.01 (0.01)
Pulse Rate		0.01** (0.002)	0.01*** (0.002)	0.01*** (0.001)	0.01*** (0.002)
Respiratory		0.01 (0.01)	0.01 (0.01)	0.01 (0.01)	0.01 (0.01)
SBP		-0.002*** (0.001)	0.00 (0.001)	-0.004*** (0.001)	-0.004*** (0.001)
AVPU		significant	significant	significant	significant
*Additional Physiological Measures*					
DBP			-0.01*** (0.002)	-0.002 (0.002)	0.00 (0.01)
SpO2%			-0.01 (0.01)	-0.001 (0.01)	-0.01 (0.01)
Physician					significant
Intercept	1.53*** (0.05)	0.22 (0.68)	1.90 (1.19)	-1.75 (1.99)	1.74 (1.92)
*R*^2^	0.09	0.12	0.13	0.27	0.42
*R*^2^ adjusted	0.09	0.11	0.13	0.26	0.34
Observations	1010	1010	1010	1010	1010

***p < 0.01,

**p < 0.05,

*p < 0.1

Note: Standard errors are in parentheses.

## Discussion

Briefly, our analysis shows that LOS is more associated with physicians’ early subjective assessments. In this analysis, MEWS only weakly explains LOS and death in models in which we exclude any vital sign and demographics. Physicians’ risk assessments are more influenced by some of the risk indicators than the MEWS system and physicians’ early assessment of risk determines the duration of a hospital stay.

Our results contradict findings from some previous studies that stressed the predictive power of MEWS [4, 12]. We believe this is because we controlled for a much greater number of variables in our analysis, and that we have a better control of in-hospital processes such as physicians’ subjective assessments, which trigger the intensity of healthcare services.

Our study hypothesizes that humans’ (physicians’) early judgments are more associated with both LOS and outcomes than several of the physiological measures. The negative association between LOS and mortality as well as the positive association between physicians’ assessment of mortality risk and LOS indicate that, like all settings in which human decision making plays an important role, early-risk measures can be more useful in guiding subsequent actions—i.e., how physicians react to initial vital signs and how such reactions may prevent catastrophic events such as mortality. Our hypothesis resonates with the results of a previous study that concludes that physicians’ and nurses’ assessments of risk of mortality is accurate [19]. While understanding the human component and that subjective assessments are important, improving the accuracy of those measures is still a necessity.

Our study focused on one specific hospital. The goal here was to ensure internal validity by focusing on a specific setting, gathering as many datapoints as possible, and being aware of all potential processes that may influence the data. Our knowledge of the hospital, the number of variables, and datapoints helped to validate our study of this specific hospital. While we do not see a major issue that may limit generalization of our study, we would like to invite other researchers to be cautious about generalizing our insights. We suggest similar studies in other hospital settings, with different governance structures, different patient demographics, and different types of physician training. In addition, we suggest more studies on effects of risk factors on patients’ perceptions and decisions in visiting emergency departments [20, 21].

Developing effective warning scores is critical for risk analysis and effective healthcare management. Our study shows that current early warnings are not effective in explaining LOS and mortality. Major feedback loop(s) exist that affect how physicians react to vital signs as well as their own subjective measures that can later compensate for large early-risk values. This comes down to the question of the purpose of early warning scores. If the scores are for predicting risk, the goal should be developing measures that provide a quick estimation of risk. However, for outcome prediction, we need more sophisticated models that embed how physicians react to different measures of risk, processes, resources, and technologies in hospitals. To conclude, we invite more studies to develop scores that can raise physician attention and improve early- assessment accuracy as well as measures that can better predict outcomes.

## Supporting Information

S1 Appendix Correlations between variables. Table A1 in this appendix shows the matrix for correlation coefficients between control variables.
(DOCX)

S1 Data Patients’ Data. Data file includes all patients’ data that are used for analysis in this paper.
(XLSX)
