# Supplementary material for: Can a Patient’s In-Hospital Length of Stay and Mortality Be Explained by Early-Risk Assessments?
Source: PLoS One. 2016 Sep 15;11(9):e0162976. doi: 10.1371/journal.pone.0162976 (PMC5024988; doi:10.1371/journal.pone.0162976)
Supplement: S1 Appendix — Table A1 in this appendix shows the matrix for correlation coefficients between control variables. (DOCX) [file pone.0162976.s001.docx]

**Appendix**

Table A1 shows the matrix for correlation coefficients between control variables.

**Table A1** Correlations between variables

|  | **Age** | **Weight** | **Height** | **BMI** | **MEWS** | **Temperature** | **Pulse Rate** | **Respiratory** | **SBP** | **DBP** | **SpO2%** | **Severity Level** | **Mortality Risk** | **LOS** |
| --- | --- | --- | --- | --- | --- | --- | --- | --- | --- | --- | --- | --- | --- | --- |
| **Age** | 1.00 |  |  |  |  |  |  |  |  |  |  |  |  |  |
| **Weight** | -0.29 | 1.00 |  |  |  |  |  |  |  |  |  |  |  |  |
| **Height** | -0.15 | 0.45 | 1.00 |  |  |  |  |  |  |  |  |  |  |  |
| **BMI** | -0.26 | 0.89 | 0.00 | 1.00 |  |  |  |  |  |  |  |  |  |  |
| **MEWS** | -0.02 | 0.01 | 0.10 | -0.04 | 1.00 |  |  |  |  |  |  |  |  |  |
| **Temperature** | 0.03 | 0.02 | -0.00 | 0.02 | -0.26 | 1.00 |  |  |  |  |  |  |  |  |
| **Pulse Rate** | -0.11 | 0.02 | 0.04 | 0.00 | 0.48 | 0.05 | 1.00 |  |  |  |  |  |  |  |
| **Respiratory** | 0.02 | 0.11 | 0.08 | 0.08 | 0.51 | -0.00 | 0.27 | 1.00 |  |  |  |  |  |  |
| **SBP** | 0.13 | 0.05 | -0.03 | 0.08 | -0.13 | 0.01 | -0.14 | -0.01 | 1.00 |  |  |  |  |  |
| **DBP** | -0.18 | 0.12 | 0.13 | 0.08 | -0.04 | -0.02 | 0.10 | -0.01 | 0.55 | 1.00 |  |  |  |  |
| **SpO2%** | -0.12 | -0.06 | 0.03 | -0.08 | -0.15 | -0.05 | -0.14 | -0.11 | 0.03 | 0.06 | 1.00 |  |  |  |
| **Severity Level** | 0.15 | -0.01 | 0.06 | -0.05 | 0.30 | -0.00 | 0.26 | 0.18 | -0.17 | -0.18 | -0.09 | 1.00 |  |  |
| **Mortality Risk** | 0.35 | -0.10 | 0.05 | -0.14 | 0.30 | -0.00 | 0.22 | 0.17 | -0.12 | -0.14 | -0.10 | 0.73 | 1.00 |  |
| **LOS** | 0.06 | -0.01 | -0.01 | 0.00 | 0.16 | 0.05 | 0.15 | 0.08 | -0.09 | -0.10 | -0.02 | 0.43 | 0.36 | 1.00 |
